# Genome-wide screen for temperature-regulated genes of the obligate intracellular bacterium, *Rickettsia typhi*

**DOI:** 10.1186/1471-2180-8-61

**Published:** 2008-04-15

**Authors:** Sheila M Dreher-Lesnick, Shane M Ceraul, M Sayeedur Rahman, Abdu F Azad

**Affiliations:** 1 Department of Microbiology and Immunology, University of Maryland, 660 W. Redwood Street, Room HH324B, Baltimore, MD 21201, USA

## Abstract

**Background:**

The ability of rickettsiae to survive in multiple eukaryotic host environments provides a good model for studying pathogen-host molecular interactions. *Rickettsia typhi*, the etiologic agent of murine typhus, is a strictly intracellular gram negative α-proteobacterium, which is transmitted to humans by its arthropod vector, the oriental rat flea, *Xenopsylla cheopis*. Thus, *R. typhi *must cycle between mammalian and flea hosts, two drastically different environments. We hypothesize that temperature plays a role in regulating host-specific gene expression, allowing *R. typhi *to survive in mammalian and arthropod hosts. In this study, we used Affymetrix microarrays to screen for temperature-induced genes upon a temperature shift from 37°C to 25°C, mimicking the two different host temperatures *in vitro*.

**Results:**

Temperature-responsive genes belonged to multiple functional categories including among others, transcription, translation, posttranslational modification/protein turnover/chaperones and intracellular trafficking and secretion. A large number of differentially expressed genes are still poorly characterized, and either have no known function or are not in the COG database. The microarray results were validated with quantitative real time RT-PCR.

**Conclusion:**

This microarray screen identified various genes that were differentially expressed upon a shift in temperature from 37°C to 25°C. Further characterization of the identified genes may provide new insights into the ability of *R. typhi *to successfully transition between its mammalian and arthropod hosts.

## Background

Rickettsiae are obligate intracellular bacteria, and are best known as the arthropod-borne disease agents of spotted and typhus fevers in humans. These diseases are prevalent throughout the world, and continue to pose potential public health problems [1]. *Rickettsia typhi *is the etiologic agent of murine typhus, a reemerging febrile illness that is endemic in coastal areas throughout the world. Its reemergence and associated epidemic outbreaks are attributed to changes in the environment and human behavior [2]. Current research efforts have focused on molecular interactions between pathogenic rickettsiae and their mammalian hosts. Studies examining arthropod-rickettsiae interactions are more scarce, despite their importance for understanding the maintenance of rickettsiae in nature [1].

Rickettsial homeostasis is continuously regulated as rickettsiae cycle between the warm-blooded vertebrate and poikilothermic invertebrate hosts. The effect of environmental temperature on gene expression is well documented for other arthropod-borne pathogens such as the agents of plague and Lyme disease, *Yersinia pestis *and *Borrelia burgdorferi*, respectively. For example, gene and protein expression studies have revealed that *Y. pestis *transmission by the cat flea, *Ctenocephalides felis*, is mediated by a murine toxin and a hemin storage protein, encoded for by *ymt and hms*, respectively [3]. Additionally, differential expression of outer surface proteins (Osps) *by Borrelia burgdorferi *has been demonstrated to be associated with colonization of the mammalian or tick host [4-7]. A temperature shift may thus also signal a change in rickettsial gene expression to overcome the drop in temperature experienced within the flea, thereby promoting survival of the rickettsiae.

The available information on rickettsial gene expression in response to temperature is limited to transcriptional characterization of individual genes [8]. DNA microarrays are widely used for transcription profiling of arthropod-borne bacteria in response to different environmental changes and provide a more comprehensive screen for differentially expressed genes. DNA microarrays have been used to determine the effect that temperature and nutrient availability on transcription profiles of *B. burgdorferi *[9-12] and *Y. pestis *[13,14] grown in cell culture.

The goal of this study was to perform a genome-wide screen for differentially transcribed *R. typhi *genes in response to a shift in temperature from 37°C to 25°C. This experimental design mimicks the temperature transition the rickettsiae may experience as they are acquired by the flea host from a rickettsemic mammal. Due to the high similarity between *R. typhi *and *R. prowazekii *genome sequences, these experiments were performed using the commercially available *R. prowazekiii *Affymetrix GeneChip. While more robust transcription profiling is achieved using same species hybridization, cross species or heterologous hybridization has been widely used to analyze global transcription profiling, and has provided reproducible, biologically relevant transcription profiles [15-18]. We hypothesize that temperature-responsive genes identified by this screen will play a key role in maintaining rickettsial homeostasis and survival as it is acquired by the flea vector. Determining the mechanisms by which rickettsiae adapt to this transition is critical for understanding rickettsial maintenance in nature.

## Results and discussion

As *R. typhi *cycles between its mammalian host and flea vector, it must adapt to different environments, including changes in temperature. In this study, we used *R. prowazekii *Affymetrix GeneChip microarrays to screen for *R. typhi *genes that are differentially expressed upon a shift from 37°C to 25°C, mimicking the transition from the mammalian host to the flea vector. The use of high density Affymetrix GeneChips which are unique in that each gene is represented by multiple, non-overlapping oligonucleotide probes [19]. This allows for reliable cross-species gene expression analysis and screening between related species [20,21]. The genomes of the two closely related typhus group rickettsiae, *R. prowazekii *and *R. typhi *have been published and annotated, and show a high degree of homology (over 80% at the DNA level) genome-wide [22,23]. Recent genomic analyses indicate that housekeeping genes, structural genes and hypothetical genes are conserved to a particularly high degree among rickettsiae, showing 90% or higher similarity between these two species [22,24].

### Microarray overview

The Affymetrix GeneChip includes multiple spots (25-mers) for all known ORFs and all intergenic regions. For this study, our aim was to screen for *R. typhi *genes that may be important for the transition from the mammal to the arthropod host. All internal hybridization controls provided with the Affymetrix GeneChips and accompanying software were verified for each run. Analysis of the presence/absence calls for the total data set (both 25°C and 37°C), indicated that transcripts for 84% of the ORFs were present, 14% absent and 2% were considered marginal calls (see Additional file 1). Of the 84% genes that were assigned have transcripts with significant presence scores, we screened for genes that possessed transcript presence scores for at least 5 out of the 6 array runs, further reducing the total number of genes used for the transcription analysis and screen to 575 (see Additional file 2). Furthermore, up to 28 *R. typhi *genes had absent transcript scores under both conditions, while 4 *R. typhi *genes had absent calls for the 37°C treatment, and 19 genes had absent transcripts scores for the 25°C treatment (see Additional file 3). Some genes with absent scores either showed no significant signal intensities or showed a higher signal intensity for the Affymetrix paired mismatch control. Strong signal values for the paired mismatch may be due to sequence differences between the *R. prowazekii *oligonucleotide sequences on the chip and the hybridizing *R. typhi *RNA sequences.

### Temperature-induced differential gene expression

Little is known about the molecular mechanisms involved in *R. typhi *transition from the mammalian host to the flea vector. Several studies note a significant increase in growth rate of rickettsiae in fleas maintained at 24°C and 30°C as compared to 18°C [25,26]. Therefore, temperature shifts may signal a change in rickettsial gene expression to acclimate to the environment experienced within the flea.

To screen for genes that may be important for this transition, we looked at *R. typhi *global transcription levels in response to a temperature shift from 37°C to 25°C. Fold change was calculated for the 575 genes analyzed using both Spotfire (Somerville, MA, 02144) and SAM software. In this screen, we set the cutoff value for differential fold expression at 1.5 [27-29]. Seventy-eight percent of these genes show no difference in expression levels upon temperature shift, while 10% of the genes are downregulated, and 12% are upregulated.

**Figure 1:**
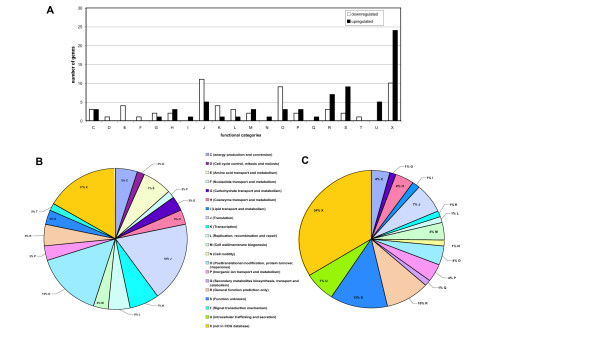
**Differentially expressed *R. typhi *genes in response to a shift in temperature from 37°C to 25°C according to gene functional group (COGs)**. Differentially expressed *R. typhi *genes were assigned their corresponding COG descriptors. (A) Comparison of number of genes per functional group found to be downregulated (white bars) versus upregulated (black bars) upon a shift down in temperature. (B) Summary of *R. typhi *genes according to functional group downregulated after a temperature shift. (C) Summary of *R. typhi *genes according to functional group upregulated after a temperature shift.

The pattern and distribution of differentially expressed genes differs for many functional categories, or COGs (Figure 1A). As expected, more genes involved in translation (COG J), transcription (COG K), posttranslational modification and protein turnover (COG O), amino acid transfer and metabolism (COG E) are downregulated upon a shift to the lower temperature (Figure 1A,B). Genes that showed a decrease in fold change expression are listed by functional category in Table 1. This correlates to slower growth rates and doubling time observed for rickettsiae at lower temperatures [30-32]. Reducing environmental temperature thus seems to trigger a reduction in metabolic and transcriptional activity of rickettsiae.

**Table 1:** Genes downregulated upon a temperature shift from 37°C to 25°C.

**Gene ID**	**Gene annotation**	**Fold Change **(exp1, exp2, exp3)
**Energy production and conversion**		
RT0261	Complex III (mitochondrial electron transport). [Rickettsia typhi str. Wilmington]	**0.5 **(0.2, 0.6, 0.9)
RT0345	NADH dehydrogenase beta subunit [Rickettsia typhi str. Wilmington]	**0.6 **(0.5, 0.6, 0.7)
RT0171	2-oxoglutarate dehydrogenase (lipoamide) E1 component [Rickettsia typhi str. Wilmington]	**0.7 **(0.5, 0.7, 0.6)
		
**Cell cycle control, mitosis and meiosis**		
RT0380	putative intracellular septation protein [Rickettsia typhi str. Wilmington]	**0.6 **(0.4, 0.9, 0.6)
		
**Amino acid transport and metabolism**		
RT0297	AtrC1-like cationic amino acid transporter protein [Rickettsia typhi str. Wilmington]	**0.6 **(0.3, 0.6, 1.8)
RT0473	cysteine desulfurase [Rickettsia typhi str. Wilmington]	**0.6 **(0.7, 0.7, 0.5)
RT0118	glutamine transport system permease protein GlnP [Rickettsia typhi str. Wilmington]	**0.6 **(0.3, 0.9, 0.4)
RT0474	cysteine desulfurase protein IscS/NifS [Rickettsia typhi str. Wilmington]	**0.6 **(0.4, 0.9, 0.6)
		
**Nucleotide transport and metabolism**		
RT0498	ribonucleotide-diphosphate reductase alpha subunit [Rickettsia typhi str. Wilmington]	**0.4 **(0.4, 0.4, 0.5)
		
**Carbohydrate transport and metabolism**		
**RT0479**	hypothetical protein RT0479 [Rickettsia typhi str. Wilmington]	**0.6 **(0.6, 0.5, 0.6)
RT0290	ribose-5-phosphate isomerase B [Rickettsia typhi str. Wilmington]	**0.6 **(1.1, 0.6, 0.3)
		
**Coenzyme transport and metabolism**		
RT0829	5-aminolevulinate synthase [Rickettsia typhi str. Wilmington]	**0.5 **(0.2, 0.5, 0.8)
**RT0501**	methylenetetrahydrofolate dehydrogenase (NADP+) [Rickettsia typhi str. Wilmington]	**0.7 **(0.6, 0.7, 0.6)
		
**Translation**		
RT0638	30S ribosomal protein S14 [Rickettsia typhi str. Wilmington]	**0.5 **(0.7, 0.6, 0.3)
RT0812	methionine aminopeptidase [Rickettsia typhi str. Wilmington]	**0.5 **(0.6, 0.5, 0.4)
RT0645	30S ribosomal protein S3 [Rickettsia typhi str. Wilmington]	**0.5 **(0.5, 0.6, 0.4)
RT0653	elongation factor Tu [Rickettsia typhi str. Wilmington]	**0.5 **(0.3, 0.6, 0.6)
RT0644	50S ribosomal protein L16 [Rickettsia typhi str. Wilmington]	**0.5 **(0.6, 0.5, 0.5)
RT0140	aspartyl/glutamyl-tRNA amidotransferase subunit B [Rickettsia typhi str. Wilmington]	**0.5 **(1.4, 0.4, 0.4)
RT0642	30S ribosomal protein S17 [Rickettsia typhi str. Wilmington]	**0.6 **(0.8, 0.6, 0.4)
RT0648	50S ribosomal protein L2 [Rickettsia typhi str. Wilmington]	**0.6 **(0.6, 0.7, 0.6)
**RT0316**	glutamyl-tRNA synthetase [Rickettsia typhi str. Wilmington]	**0.6 **(0.5, 0.8, 0.6)
RT0372	prolyl-tRNA synthetase [Rickettsia typhi str. Wilmington]	**0.7 **(0.4, 0.7, 0.9)
RT0606	isoleucyl-tRNA synthetase [Rickettsia typhi str. Wilmington]	**0.7 **(0.4, 0.8, 0.7)
		
**Transcription**		
RT0129	DNA-directed RNA polymerase beta subunit [Rickettsia typhi str. Wilmington]	**0.5 **(0.3, 0.5, 0.8)
RT0130	DNA-directed RNA polymerase beta' subunit [Rickettsia typhi str. Wilmington]	**0.7 **(0.6, 0.6, 0.9)
**RT0152**	transcription antitermination protein NusB [Rickettsia typhi str. Wilmington]	**0.7 **(0.6, 0.7, 0.6)
RT0061	transcriptional activator protein CzcR [Rickettsia typhi str. Wilmington]	**0.6 **(0.5, 0.7, 0.6)
		
**Replication, recombination and repair**		
RT0706	DNA ligase [Rickettsia typhi str. Wilmington]	**0.4 **(0.3, 0.3, 0.6)
RT0228*	dinucleoside polyphosphate hydrolase [Rickettsia typhi str. Wilmington]	**0.5 **(0.5, 0.5, 0.4)
**RT0805**	tyrosine recombinase [Rickettsia typhi str. Wilmington]	**0.6 **(0.7, 0.6, 0.6)
		
**Cell wall/membrane biogenesis**		
RT0393*	hypothetical protein RT0393 [Rickettsia typhi str. Wilmington]	**0.6 **(0.6, 0.6, 0.6)
RT0326	probable glycosyltransferase [Rickettsia typhi str. Wilmington]	**0.7 **(0.6, 0.8, 0.4)
		
**Posttranslational modification, protein turnover, chaperones**		
RT0319*	hypothetical protein RT0319 [Rickettsia typhi str. Wilmington]	**0.5 **(0.3, 0.6, 0.3)
RT0617	chaperonin GroEL [Rickettsia typhi str. Wilmington]	**0.5 **(0.4, 0.4, 1.0)
RT0191	co-chaperone HscB [Rickettsia typhi str. Wilmington]	**0.5 **(0.7, 0.7, 0.2)
RT0828*	heat shock protein 90 [Rickettsia typhi str. Wilmington]	**0.5 **(0.3, 0.6, 0.5)
RT0318	thioredoxin peroxidase I [Rickettsia typhi str. Wilmington]	**0.5 **(0.6, 0.8, 0.4)
RT0111	protease activity modulator protein HflK [Rickettsia typhi str. Wilmington]	**0.6 **(0.8, 0.4, 0.6)
RT0476	Glutathione S-alkyltransferase. [Rickettsia typhi str. Wilmington]	**0.6 **(0.2, 0.7, 1.1)
RT0618*	co-chaperonin GroES [Rickettsia typhi str. Wilmington]	**0.6 **(0.5, 0.5, 0.7)
RT0176	molecular chaperone DnaK [Rickettsia typhi str. Wilmington]	**0.6 **(0.4, 0.6, 1.1)
		
**Inorganic ion transport and metabolism**		
RT0612	zinc/manganese ABC transporter permease protein [Rickettsia typhi str. Wilmington]	**0.6 **(0.3, 1.1, 0.6)
RT0258	hypothetical protein RT0258 [Rickettsia typhi str. Wilmington]	**0.6 **(0.5, 0.9, 0.6)
		
**General function prediction only**		
RT0320*	hypothetical protein RT0320 [Rickettsia typhi str. Wilmington]	**0.6 **(0.5, 0.5, 0.6)
RT0431	hypothetical protein RT0431 [Rickettsia typhi str. Wilmington]	**0.6 **(0.4, 0.6, 1.2)
RT0035	GTP-binding protein [Rickettsia typhi str. Wilmington]	**0.6 **(0.3, 0.9, 1.2)
		
**Function unknown**		
RT0471	iron-binding protein IscA/HesB [Rickettsia typhi str. Wilmington]	**0.5 **(0.4, 0.7, 0.6)
RT0519*	rickettsial conserved hypothetical protein [Rickettsia typhi str. Wilmington]	**0.6 **(0.7, 0.7, 0.4)
		
**Signal transduction mechanism**		
RT0550	nitrogen assimilation regulatory protein NtrX [Rickettsia typhi str. Wilmington]	**0.6 **(0.5, 0.7, 0.6)
		
**not in COG database**		
RT0259	heme exporter protein B [Rickettsia typhi str. Wilmington]	**0.5 **(0.6, 0.8, 0.3)
RT0077*	nucleoside diphosphate kinase [Rickettsia typhi str. Wilmington]	**0.5 **(0.6, 0.5, 0.5)
**RT0874**	coproporphyrinogen III oxidase [Rickettsia typhi str. Wilmington]	**0.5 **(0.7, 0.7, 0.3)
RT0325*	rickettsial conserved hypothetical protein [Rickettsia typhi str. Wilmington]	**0.6 **(0.6, 0.8, 0.4)
RT0426	rickettsial conserved hypothetical protein [Rickettsia typhi str. Wilmington]	**0.6 **(0.2, 0.6, 1.5)
RT0485	cell surface antigen [Rickettsia typhi str. Wilmington]	**0.6 **(1.1, 0.7, 0.2)
RT0686	rickettsial conserved hypothetical protein [Rickettsia typhi str. Wilmington]	**0.6 **(0.7, 0.6, 0.5)
RT0126	50S ribosomal protein L1 [Rickettsia typhi str. Wilmington]	**0.6 **(0.6, 1.0, 0.6)
RT0102	hypothetical protein RT0102 [Rickettsia typhi str. Wilmington]	**0.6 **(0.6, 0.8, 0.5)
RT0701	possible beta-glucosidase [Rickettsia typhi str. Wilmington]	**0.6 **(0.8, 0.6, 0.5)

Genes are grouped by functional categories. Gene IDs with asterisks and/or bold are statistically significant (see Methods). Fold change values in bold were obtained by comparing the normalized mean signal intensities of all three independent experiments using the Spotfire analysis program. Immediately following these values are the fold change values for each individual experiment (experiment 1, experiment 2 and experiment 3, respectively).

However, while metabolic activity and overall transcription may be reduced, genes belonging to other functional categories are upregulated upon a shift to 25°C (Figure 1A,C). These genes may be important for *R. typhi *homeostasis in the insect vector. Functional categories of particular interest include intracellular trafficking and secretion (COG U), cell wall and membrane biogenesis (COG M), and genes that are not fully characterized or whose functions are unknown (COGs R, S and X). Gene IDs and corresponding fold change are listed by functional category in Table 2. It is interesting to note that 57% of the genes found to be upregulated either have no clearly defined function (COGs S and R) or are not assigned to any functional categories at all (COG X) (Figure 1B). Further characterization of these genes and their corresponding protein products will be important to better understand the biology of rickettsiae as they cycle between arthropod and mammalian hosts.

**Table 2:** Genes upregulated upon a temperature shift from 37°C to 25°C.

**Gene ID**	**Gene annotation**	**Fold Change **(exp1, exp2, exp3)
**Energy production and conversion**		
RT0308	hypothetical protein RT0308 [Rickettsia typhi str. Wilmington]	**1.5 **(2.2, 1.4, 1.0)
RT0346	NADH dehydrogenase alpha subunit [Rickettsia typhi str. Wilmington]	**1.6 **(1.2, 2.0, 1.8)
RT0419*	succinyl-CoA synthetase alpha subunit [Rickettsia typhi str. Wilmington]	**1.9 **(2.1, 1.7, 1.8)
		
**Carbohydrate transport and metabolism**		
RT0478*	pyruvate phosphate dikinase [Rickettsia typhi str. Wilmington]	**1.6 **(1.9, 1.5, 1.6)
		
**Coenzyme transport and metabolism**		
RT0867	lipoyltransferase [Rickettsia typhi str. Wilmington]	**1.6 **(1.3, 1.9, 1.7)
RT0530*	3-octaprenyl-4-hydroxybenzoate carboxy-lyase [Rickettsia typhi str. Wilmington]	**2.0 **(2.0, 1.5, 2.3)
RT0675*	ubiquinone/menaquinone biosynthesis methyltransferase [Rickettsia typhi str. Wilmington]	**2.8 **(3.7, 2.4, 2.2)
		
**Lipid transport and metabolism**		
RT0676*	hypothetical protein RT0676 [Rickettsia typhi str. Wilmington]	**1.8 **(3.0, 1.5, 1.6)
		
**Translation**		
RT0337	16S rRNA-processing protein [Rickettsia typhi str. Wilmington]	**1.9 **(2, 1.3, 2.1)
RT0604*	30S ribosomal protein S21 [Rickettsia typhi str. Wilmington]	**2.2 **(2.1, 2.6, 2.2)
RT0014	NTP polymerase. [Rickettsia typhi str. Wilmington]	**2.3 **(5.6, 1.1, 1.5)
RT0870*	50S ribosomal protein L33 [Rickettsia typhi str. Wilmington]	**2.3 **(2.3, 1.9, 2.5)
RT0038	50S ribosomal protein L28 [Rickettsia typhi str. Wilmington]	**2.5 **(4.1, 1.7, 1.3)
RT0484	hypothetical protein RT0484 [Rickettsia typhi str. Wilmington]	**1.6 **(2.0, 1.6, 1.4)
		
**Replication, recombination and repair**		
RT0339	exodeoxyribonuclease VII small subunit [Rickettsia typhi str. Wilmington]	**1.6 **(1.8,1.0,1.9)
		
**Cell wall/membrane biogenesis**		
RT0815	possible outer surface protein [Rickettsia typhi str. Wilmington]	**1.6 **(2.3, 1.2, 1.2)
RT0083	hypothetical protein RT0083 [Rickettsia typhi str. Wilmington]	**1.7 **(2.8, 1.1, 1.1)
RT0694	hypothetical protein RT0694 [Rickettsia typhi str. Wilmington]	**1.9 **(2.6, 2.0, 1.3)
		
**Cell motility**		
RT0457*	rickettsial conserved hypothetical protein [Rickettsia typhi str. Wilmington]	**1.9 **(2.1, 1.9, 1.5)
		
**Posttranslational modification, protein turnover, chaperones**		
RT0620	HSP-70 cofactor [Rickettsia typhi str. Wilmington]	**1.6 **(1.7, 1.5, 1.5)
RT0416	SsrA-binding protein [Rickettsia typhi str. Wilmington]	**1.6 **(1.5, 2.5, 1.0)
RT0195*	probable ATP binding cassette transporter [Rickettsia typhi str. Wilmington]	**2.8 **(2.1, 2.5, 5.2)
		
**Inorganic ion transport and metabolism**		
RT0168	probable periplasmic divalent cation tolerance protein CutA [Rickettsia typhi str. Wilmington]	**1.5 **(1.3, 1.2, 2.1)
RT0013	probable zinc/manganese ABC transporter substrate binding protein	**1.8 **(2.5, 1.6, 1.3)
RT0523	superoxide dismutase [Rickettsia typhi str. Wilmington]	**1.8 **(1.8, 1.5, 2.0)
		
**Secondary metabolites biosynthesis, transport and catabolism**		
RT0043*	hypothetical protein RT0043 [Rickettsia typhi str. Wilmington]	**1.6 **(1.8, 1.6, 1.3)
		
**General function prediction only**		
RT0576	Sco2-like protein [Rickettsia typhi str. Wilmington]	**1.6 **(1.7, 1.5, 1.5)
RT0012	hypothetical protein RT0012 [Rickettsia typhi str. Wilmington]	**1.6 **(2.2, 1.3, 1.3)
RT0138	hypothetical protein RT0138 [Rickettsia typhi str. Wilmington]	**1.6 **(1.4, 1.0, 2.2)
RT0674	ubiquinone biosynthesis protein [Rickettsia typhi str. Wilmington]	**1.6 **(1.3, 1.4, 2.4)
RP031	no corresponding annotation for *R. typhi*	**1.8 **(1.1, 2.9, 1.4)
RP679	no corresponding annotation for *R. typhi*	**2.1 **(1.7, 1.9, 3.3)
RT0362*	Sec7-domain containing protein transport protein [Rickettsia typhi str. Wilmington]	**2.6 **(3.3, 5.6, 1.7)
		
**Function unknown**		
RT0741	rickettsial conserved hypothetical protein [Rickettsia typhi str. Wilmington]	**1.5 **(1.0, 2.0, 1.7)
RT0716*	rickettsial conserved hypothetical protein [Rickettsia typhi str. Wilmington]	**1.6 **(1.6, 1.8, 1.5)
RT0551	rickettsial conserved hypothetical protein [Rickettsia typhi str. Wilmington]	**1.7 **(1.4, 1.8, 1.6)
RP167	no corresponding annotation for *R. typhi*	**1.9 **(2.9, 1.7, 1.4)
**RT0160**	rickettsial conserved hypothetical protein [Rickettsia typhi str. Wilmington]	**2.0 **(2.4, 1.3, 2.1)
RT0222	hypothetical protein RT0222 [Rickettsia typhi str. Wilmington]	**2.0 **(2.8, 1.2, 1.7)
RT0069	iron-binding protein IscA/HesB [Rickettsia typhi str. Wilmington]	**2.1 **(2.2, 1.6, 2.1)
RT0743	rickettsial conserved hypothetical protein [Rickettsia typhi str. Wilmington]	**2.1 **(2.6, 1.1, 2.3)
RT0541	hypothetical protein RT0541 [Rickettsia typhi str. Wilmington]	**2.4 **(2.3, 3.5, 2.3)
		
**Intracellular trafficking and secretion**		
RT0053	protein-export membrane protein [Rickettsia typhi str. Wilmington]	**1.6 **(1.8, 2.2, 0.9)
RT0164	signal recognition particle protein [Rickettsia typhi str. Wilmington]	**1.7 **(1.8, 1.2, 1.9)
RT0280	VirB8-like protein of the Type IV secretion system [Rickettsia typhi str. Wilmington]	**1.8 **(2.6, 1.4, 1.6)
RT0771*	VirB4 protein precursor [Rickettsia typhi str. Wilmington]	**1.9 **(1.9, 2.1, 1.7)
RT0278*	VirB8-like protein of type IV secretion system [Rickettsia typhi str. Wilmington]	**2.6 **(3.6, 2.8, 1.8)
		
**not in COG**		
RT0351	rickettsial conserved hypothetical protein [Rickettsia typhi str. Wilmington]	**1.5 **(1.8, 1.0, 1.5)
RT0767*	rickettsial conserved hypothetical protein [Rickettsia typhi str. Wilmington]	**1.5 **(1.6, 1.3, 1.7)
RT0861	hypothetical protein RT0861 [Rickettsia typhi str. Wilmington]	**1.6 **(1.2, 2.1, 1.4)
RT0080	rickettsial conserved hypothetical protein [Rickettsia typhi str. Wilmington]	**1.6 **(2.2, 1.0, 1.3)
**RT0693**	bicyclomycin resistance protein [Rickettsia typhi str. Wilmington]	**1.6 **(1.1, 1.8, 1.9)
RT0156	rickettsial conserved hypothetical protein [Rickettsia typhi str. Wilmington]	**1.6 **(2.4, 1.2, 1.5)
RT0461	rickettsial conserved hypothetical protein [Rickettsia typhi str. Wilmington]	**1.6 **(1.5, 1.6, 1.7)
RT0097	hypothetical protein RT0097 [Rickettsia typhi str. Wilmington]	**1.8 **(2.5, 1.4, 1.2)
RP084	no corresponding annotation for *R. typhi*	**1.8 **(2.7, 1.6, 1.5)
RT0052	190 kDa antigen precursor [Rickettsia typhi str. Wilmington]	**1.8 **(1.2, 1.6, 2.5)
RT0286	rickettsial conserved hypothetical protein [Rickettsia typhi str. Wilmington]	**1.9 **(1.4, 1.6, 3.3)
RT0443	50S ribosomal protein L36 [Rickettsia typhi str. Wilmington]	**1.9 **(1.9, 1.4, 2.2)
RT0206	rickettsial conserved hypothetical protein [Rickettsia typhi str. Wilmington]	**2.0 **(2.4, 1.6, 1.8)
RT0246*	UDP-3-O- [3-hydroxymyristoyl] N-acetylglucosamine deacetylase [Rickettsia typhi str. Wilmington]	**2.1 **(2.5, 1.8, 2.0)
RT0382	hypothetical protein RT0382 [Rickettsia typhi str. Wilmington]	**2.2 **(2.8, 2.0, 1.8)
RT0279	rickettsial conserved hypothetical protein [Rickettsia typhi str. Wilmington]	**2.2 **(5.2, 1.3, 1.3)
RT0710*	rickettsial conserved hypothetical protein [Rickettsia typhi str. Wilmington]	**2.3 **(2.8, 1.7, 2.2)
RT0269	rickettsial conserved hypothetical protein [Rickettsia typhi str. Wilmington]	**2.3 **(1.8, 6.6, 1.3)
RT0692*	rickettsial conserved hypothetical protein [Rickettsia typhi str. Wilmington]	**2.7 **(3.4, 1.7, 2.6)
RT0806*	rickettsial conserved hypothetical protein [Rickettsia typhi str. Wilmington]	**2.7 **(2.3, 2.6, 3.3)
RT0709*	rickettsial conserved hypothetical protein [Rickettsia typhi str. Wilmington]	**2.9 **(2.5, 3.1, 3.1)
RT0071	rickettsial conserved hypothetical protein [Rickettsia typhi str. Wilmington]	**3.2 **(2.9, 1.5, 6.0)
RT0268*	rickettsial conserved hypothetical protein [Rickettsia typhi str. Wilmington]	**6.2 **(9.0, 6.1, 4.0)

Genes are grouped by functional categories. Gene IDs with asterisks and/or bold are statistically significant (see Methods). Fold change values in bold were obtained by comparing the normalized mean signal intensities of all three independent experiments using the Spotfire analysis program. Immediately following these values are the fold change values for each individual experiment (experiment 1, experiment 2 and experiment 3, respectively).

Temperature-induced differential gene expression has been shown to play a role in other arthropod-borne bacteria, most notably *Borrelia burgdorferi *[9-12] and *Yersinia pestis *[13,14]. In these systems, temperature-induced differential gene expression is linked to host-specific gene expression, which in turn allows for effective transition into the new host environment. Revel *et al*. [12] investigated differential transcription of *B. burgdorferi *grown in conditions mimicking unfed and fed ticks. They observed temperature-dependent down-regulation of genes involved in cell envelope synthesis, transport and binding [12]. In another study, temperature alone is reported to modulate the expression of hemolytic proteins, cell envelope proteins, transporter proteins, biosynthesis proteins and multiple hypothetical proteins of unknown function [10]. Temperature shifts are also reported to affect global transcription patterns of *Y. pestis*. [13,14]. Indeed, Han *et al*. [13] found 15% of *Y. pestis *genes were differentially expressed in response to a shift in temperature, with 61% of the differentially expressed genes being up-regulated at 26°C relative to 37°C. As expected, all genes in the hemin storage locus (*hms*), which play an important role in *Y. pestis *transmission by the flea vector, were found to be up-regulated at 26°C *in vitro *[13]. The *hms *regulator (*hmsT*), in turn was up-regulated at 37°C, providing further evidence that temperature is an important factor controlling host-specific gene expression.

### Heat shock genes and chaperones

Multiple heat shock genes were downregulated upon a shift to the lower temperature. These include heat shock proteins GroES, and heat shock protein 90, and hypothetical protein RT0319 (Table 1). Nine out of 10 differentially regulated genes within this functional group (Cog O, posttranslational modification, protein turnover, chaperones) were downregulated upon a reduction in temperature (Tables 1 and 2). Heat shock proteins including GroESL are known to play important roles in the infection cycle of many bacteria by maintaining proper protein folding in response to environmental stresses including heat shock, low pH and oxidative stress [33], as well as maintaining proper protein folding under optimal growth conditions [34]. In rickettsiae, the *groESL *operon is bicistronically transcribed. Rickettsiae appear to constitutively transcribe *groESL *in mammalian hosts [35], with GroESL showing strong antigenic properties [36]. Similarly, facultative intracellular bacteria show transcription of GroESL within mammalian host cells [37,38], showing increased levels of transcription immediately following entry into mammalian cells [39]. In other bacteria, heat shock genes from this protein family are generally found to be upregulated upon a shift up in temperature [29,40]. It is not surprising therefore, that in our study, these genes were downregulated upon a temperature shift from 37°C to 25°C. The decrease in heat shock gene transcripts may result from the reduction in overall transcription, translation (Figure 1) and growth observed for rickettsiae at these lower temperatures [30-32]. Further transcriptional and proteomic analyses of rickettsial heat shock proteins in different host environments are needed to determine the role of these proteins in rickettsial survival and pathogenesis in either the mammalian or arthropod host.

### Intracellular trafficking and secretion

Soon after uptake by the host cell, rickettsiae lyse the phagosome and replicate in the host cell cytoplasm [41]. Phagosomal escape by typhus group rickettsiae is thought to be facilitated by secreted membranolytic proteins encoded by *pld *and *tlyC *[42]. In many bacterial systems, virulence is often associated with bacterial secretory proteins and protein secretion systems. In our study, we found several genes encoding proteins for the type IV secretion system, RT0280, RT0771 and RT0278, to be upregulated upon a temperature shift from 37°C to 25°C (Table 2). Rickettsial genomes possess genes that encode proteins for the type IV secretion system similar to those identified and well characterized in *Agrobacterium tumefaciens *[22,43,44]. Bacterial type IV secretion systems are functionally diverse and are known to allow for interbacterial DNA transfer, as is the case with the ComB system of *Helicobacter pylori*. Additionally, type IV secretion systems mediate secretion of effector proteins such as RalF, a virulence factor essential for intracellular survival in *Legionella *[43,45,46]. While rickettsiae contain genes that encode the type IV secretory apparatus, the role of the rickettsial type IV secretory apparatus and its possible effector proteins in rickettsial survival and pathogenesis remains unknown.

### Energy and metabolism

This screen also identified genes known to be involved in transport and metabolism of amino acids, nucleotides, and carbohydrates (Figure 1). This pattern is in concordance with the number and pattern of downregulated versus upregulated genes that are involved in transcription, translation and replication, with more *R. typhi *genes observed to be downregulated in these functional groups (Figure 1). Interestingly, RT0118, a gene encoding a glutamate transport system permease protein showed a 1.5 fold downregulation upon the temperature shift (Table 1). Bovarnick and Snyder [47] were the first to show the importance of glutamate in augmenting rickettsial respiration. Williams and Weiss [48] expanded the study on the effect of glutamate on rickettsial metabolism and found that glutamate and glutamine were the most efficient nutrients for ATP production in *R. typhi*. Downregulation of genes involved in various metabolic processes suggests that *R. typhi *reduces its metabolic activity soon after a shift down in temperature.

### Validation of microarray results

**Figure 2:**
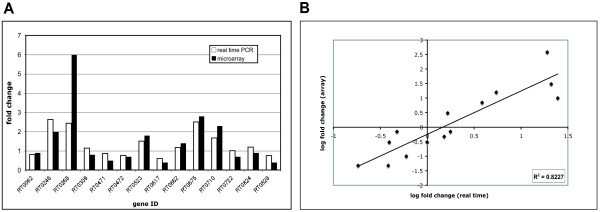
**Validation of microarray data and comparison with real time qRT-PCR data**. (A) Comparison of real time (white bars) and microarray (black bars) fold change results for 14 select *R. typhi *genes listed in Additional file 6. Fold change ratios represent the difference in transcript abundance/signal for these genes post temperature shift from 37°C to 25°C. (B) Correlation analysis of microarray and real time transcript measurements for the 14 select *R. typhi *genes mentioned above. The real time qRT-PCR log2 values were plotted against the microarray log_2 _values. The correlation coefficient (R^2^) between the two datasets is 0.8227.

Real time quantitative RT-PCR was used to validate our microarray screen. Temperature shift studies were repeated in triplicate, and *R. typhi *RNA was isolated as described (see Materials and Methods). Transcription data were obtained for 15 genes representing upregulated, downregulated and unchanged genes (Figure 2). While the fold change values obtained from our real time analysis showed some variation compared to the fold change values obtained in our arrays, the general trends remain consistent (Figure 2A). Log fold change of relative transcript abundance between the two temperature conditions was calculated and plotted against the average log fold change values obtained from the arrays (Figure 2B). We found a positive correlation (R^2 ^= 0.8227) between the values obtained from the two techniques. The real time qRT-PCR analysis thus supports the trends observed in our array results, but demonstrates a need to verify transcription trends for individual genes during follow up studies.

### *R. typhi *genes not present on array

**Figure 3:**
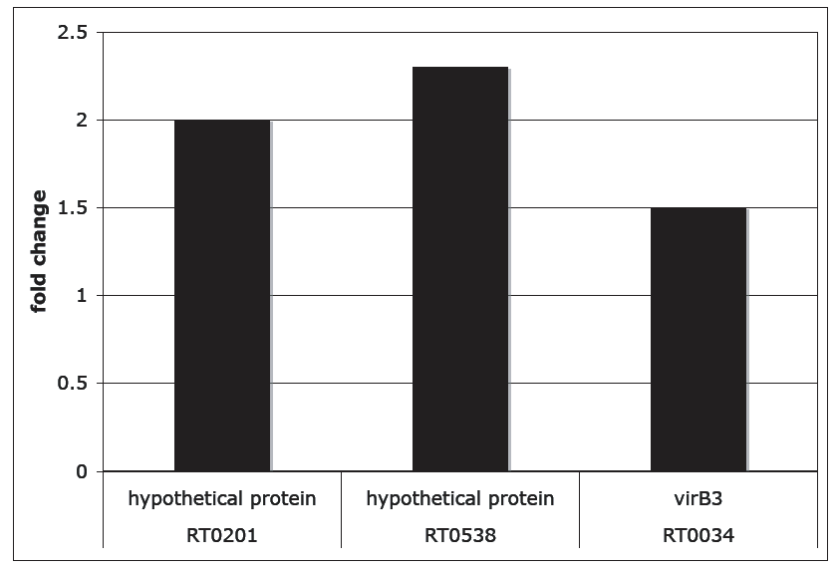
**Differentially expressed *R. typhi *genes not present on the array**. *R. typhi *genes not present on the array were screened using real time qRT-PCR, and fold change was calculated.

We screened 13 *R. typhi *genes that were not present on the *R. prowazekii *GeneChip for differential gene expression upon a temperature shift using real time qRT-PCR. Of these, only 3 genes were differentially expressed (Figure 3). Interestingly, another type IV secretory component, *virb3*, was found to be upregulated, a pattern that is also observed in the arrays (Table 2).

## Conclusion

To date, little is known about the molecular mechanisms underlying rickettsial survival in its eukaryotic host, in part due to the lack of genetic manipulation tools and the strict intracellular lifestyle of rickettsiae. The completion of several *Rickettsia *genomes allows us to use genomic and bioinformatics tools to study rickettsia-host interactions. While comparative genomics can yield useful information regarding what genetic machinery is present, it does not provide any insight into timing and regulation of gene expression in its different eukaryotic hosts.

In this study, we used commercially available *R. prowazekii *Affymetrix GeneChip arrays to screen for temperature-regulated *R. typhi *genes that may play a role in transitioning from the mammalian host to the flea vector. To our knowledge, this screen is the first global transcriptome analysis of any rickettsial species. Temperature-responsive genes belonged to multiple functional categories. Several genes grouped in functional categories for transcription, translation, metabolism and posttranslational modification (heat shock genes) were found to be downregulated upon a shift down in temperature. Alternately, genes encoding proteins involved in intracellular trafficking and secretion and multiple genes of unknown function (hypothetical proteins) were upregulated post temperature shift. Further characterization of the identified temperature-responsive genes will allow us to better understand how *R. typhi *successfully regulates transcription to maintain homeostasis and survive in drastically changing environments.

## Materials and methods

### *R. typhi *growth conditions and purification

Low subculture mouse fibroblast cells (L929, ATCC CCL1) were grown in Dulbecco's Modified Eagle's Medium (DMEM) supplemented with 5% FBS at 37°C and 5% CO_2 _in 150 cm^2 ^vented lid flasks. *R. typhi *(Wilmington strain) was routinely quantified using the Baclight Live/dead assay (Molecular probes, Eugene, OR). When the L929 cells reached 80% confluency, they were infected with *R. typhi *at an MOI of 10. Infected cells were kept at 37°C for 4 days to allow establishment of the infection. On day 4, half of the *R. typhi *cultures were harvested while the remaining cultures were switched to 25°C and harvested 2 hours post temperature shift. Multiple flasks were pooled for each temperature to attain enough rickettsiae for RNA extraction. The experiment was performed in triplicate.

To harvest rickettsia, the infected L929 cells were washed with SPG (sucrose phosphate glucose) buffer, then scraped and resuspended in 3 mL of fresh SPG buffer. The lysates were then sonicated at setting 6.5 twice for 30 seconds using a Sonic Dismembrenator (Fischer Scientific). To remove large host cell material, lysates were centrifuged at 1000×g for 5 minutes. The supernatants (containing rickettsiae) were then placed over a 25% renografin bed and centrifuged at 14,000×g for 10 minutes. The pellets were washed twice in SPG buffer and centrifuged at 14,000× g to collect the pellet, which was processed for RNA isolation as described below.

### RNA isolation and processing

Total RNA from the rickettsial pellet was isolated using the Qiagen RNAeasy Micro Kit (Qiagen, Valencia, CA) following manufacturer's protocol. RNA was DNAse treated for an additional 30 minutes to ensure complete digestion of rickettsial DNA. Rickettsial RNA was then eluted in RNAse/DNAse free water. RNA samples were then run through the Agilent 2100 Bioanalyzer (Agilent Technologies, Inc.) to check for purity and integrity.

### Microarray screen

*R. typhi *RNA from the two temperature treatments was used to generate biotin-labeled cDNA, which was then exposed to the commercially available *R. prowazekii *whole genome Affymetrix GeneChip. The Affymetrix GeneChips were processed by the University of Maryland Baltimore Biopolymer and Genomics Core facility according to standard Affymetrix protocols and manuals for prokaryotic samples and arrays [49]. Each temperature treatment was run in triplicate to account for technical and intersample variation for a total of 6 microarray runs (see Additional file 4).

### Microarray data analysis

Expression signal data were read and processed with the Affymetrix GCOS software program using default settings. All internal hybridization controls were verified for each run as described in the GCOS manual. Assembled expression data were then viewed and analyzed using Spotfire (Somerville, MA, 02144), and SAM (Statistical Analysis of Microarrays) [50,51]. In this study, expression data for intergenic regions were not included in the analysis. All genes present on the array were assigned absent and present scores by the GCOS software program. Only genes that had at least 5 (out of 6) present scores for all array runs were included for further analysis.

To look for differences in expression levels in response to a temperature shift, array data were normalized according to default settings in Spotfire, where raw values were normalized to the column mean. Fold change in gene expression was determined by dividing average signal values for each gene at 25°C by their corresponding average signal values at 37°C. Data were also analyzed using SAM to further evaluate significant fold change in expression values. The two analysis programs were not in complete concordance, but did show some overlap (see Additional file 5). All genes that show statistical significance for differential expression are highlighted by asterisk or in bold in Tables 1 and 2. Gene IDs followed by an asterisk were found to be significantly downregulated or upregulated by the SAM software program for Delta = 0.293. Gene IDs in bold were found to be significantly downregulated or upregulated with a p value of p < 0.05 using the t-test/ANOVA provided with the Spotfire microarray analysis software program. All p values as well as fold change values for each individual experiment are listed in Additional file 2.

Genes present and annotated on the *R. prowazekii *Affymetrix array were matched with the corresponding *R. typhi *gene ID and annotations (see Additional file 2) listed in the NCBI database. For functional gene classification, each gene was also assigned their COG descriptor (cluster of orthologous groups) as determined by the NCBI database.

### Real time quantitative RT-PCR

Temperature shift experiments were repeated in triplicate for validation studies. *R. typhi *were cultured, isolated and RNA purified as described above. Primers were designed using MacVector (see Additional file 6) and the guidelines set forth in Stratagene's Guide to Quantitative PCR (QPCR Guide SMO90106). After DNAse treatment, RNA was checked for genomic DNA (gDNA) contamination by PCR using standard conditions. cDNA was synthesized with random hexamers from gDNA-free RNA using Superscript III First Strand Synthesis Supermix Kit for qRT-PCR (Invtrogen, Carlsbad, CA, 92008) according to the manufacturer. qPCR reactions were set up according to manufacturer's protocol for the SYBRGreener Supermix for qPCR (Invitrogen) using *R. typhi *cDNA from 25°C and 37°C as our templates. Reactions were run on a Stratagene Mx3000p thermocycler (Stratagene, La Jolla, CA, 92037). PCR efficiencies for each primer pair was determined using LinReg PCR [52], which was then incorporated into the normalization calculations using the qgene software program [53]. The *R. typhi *gene *rpsL *was used as the endogenous housekeeping gene for normalization. We took the average of the normalized values to calculate fold change in gene expression between the two treatments. The real time data was then correlated to the corresponding array expression data by taking the log fold change of the two and plotting them against each other.

### Screening for differential expression of *R. typhi *genes not on array

The *R. typhi *genome contains 24 genes that were not present on the array. Thirteen of the 24 genes were screened using real time qRT-PCR as described above. The remaining ORFs were too small for efficient primer design and amplification.

## Competing interests

The author(s) declare that they have no competing interests.

## Authors' contributions

SMDL and SMC designed the research project and performed the experiments. SMDL analyzed the data and drafted the manuscript. MSR and AFA participated in the design of the study. All authors contributed to the writing and preparation of the manuscript. All authors read and approved the manuscript.

## Supplementary Material

Additional file 1 **Analysis of the Affymetrix presence and absent calls for genes on the array**. Figure showing overview of all genes on the array sorted by the Affymetrix assigned presence and absence scores for all 6 replicates. Click here for file

Additional file 2 **Final list of genes that were screened for differential expression patterns in response to a shift down in temperature**. This excel file is a comprehensive list for the genes selected for continued analysis for this study. It contains the gene IDs, annotations, COG descriptors, fold change values and p values. Click here for file

Additional file 3 **List of genes with absent calls on all array runs**. This excel file contains a list of all the genes that showed low or no signal intensities and were assigned absent calls for all replicates by the Affymetrix GCOS software program. Click here for file

Additional file 4 **Raw array data**. This excel file contains the raw data for all 6 arrays. Click here for file

Additional file 5 **Venn Diagram outlining overlap in fold change analysis using Spotfire and SAM**. A. Venn diagram showing number of genes with significant downregulated fold change using SAM and Spotfire. B. Venn diagram showing number of genes with significant upregulated fold change using SAM and Spotfire. Click here for file

Additional file 6 **List of primers used for Real Time qRT-PCR validation**. This excel file contains the list of genes analyzed for Real Time qRT-PCR and the corresponding primer sequences. Click here for file
