# Supplementary figures and images for: Genome-wide screen for temperature-regulated genes of the obligate intracellular bacterium, Rickettsia typhi
Source: BMC Microbiol. 2008 Apr 15;8:61. doi: 10.1186/1471-2180-8-61 (PMC2335108; doi:10.1186/1471-2180-8-61)

## Slide 1
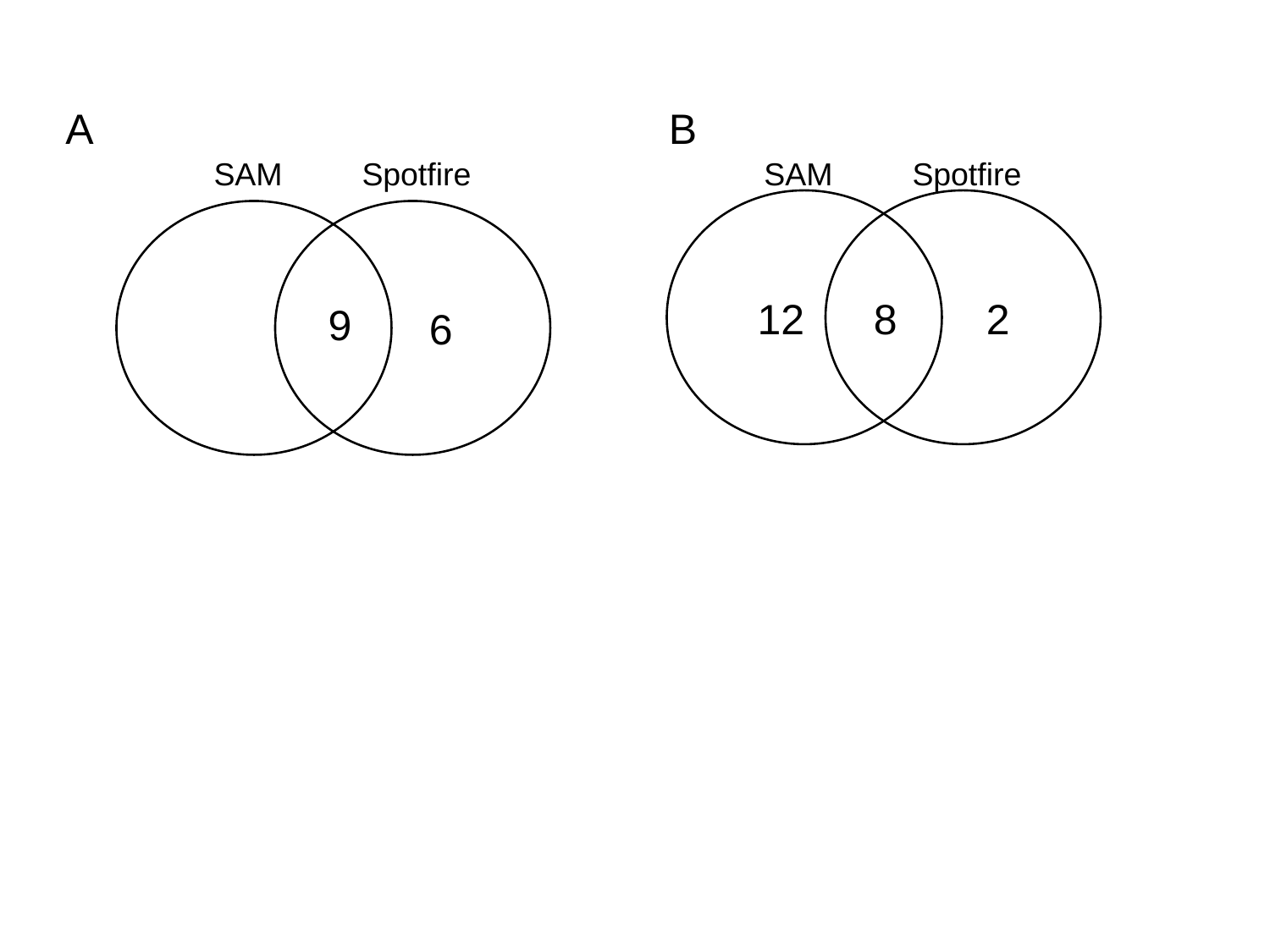

A
SAM
Spotfire
B
SAM
Spotfire
12
8
2
9
6

Supplement: Additional file 5 — Venn Diagram outlining overlap in fold change analysis using Spotfire and SAM. A. Venn diagram showing number of genes with significant downregulated fold change using SAM and Spotfire. B. Venn diagram showing number of genes with significant upregulated fold change using SAM and Spotfire. [file 1471-2180-8-61-S5.ppt]
